# Mobile fNIRS for exploring inter-brain synchrony across generations and time

**DOI:** 10.3389/fnrgo.2023.1260738

**Published:** 2024-01-03

**Authors:** Ryssa Moffat, Courtney E. Casale, Emily S. Cross

**Affiliations:** ^1^Social Brain Sciences, ETH Zurich, Zurich, Switzerland; ^2^School of Psychological Sciences, Macquarie University, Sydney, NSW, Australia

**Keywords:** fNIRS, hyperscanning, intergenerational, longitudinal, interpersonal neural synchrony, inter-brain synchrony

## Abstract

While still relatively rare, longitudinal hyperscanning studies are exceptionally valuable for documenting changes in inter-brain synchrony, which may in turn underpin how behaviors develop and evolve in social settings. The generalizability and ecological validity of this experimental approach hinges on the selected imaging technique being mobile–a requirement met by functional near-infrared spectroscopy (fNIRS). fNIRS has most frequently been used to examine the development of inter-brain synchrony and behavior in child-parent dyads. In this position paper, we contend that dedicating attention to longitudinal *and* intergenerational hyperscanning stands to benefit the fields of social and cognitive neuroscience more broadly. We argue that this approach is particularly relevant for understanding the neural mechanisms underpinning intergenerational social dynamics, and potentially for benchmarking progress in psychological and social interventions, many of which are situated in intergenerational contexts. In line with our position, we highlight areas of intergenerational research that stand to be enhanced by longitudinal hyperscanning with mobile devices, describe challenges that may arise from measuring across generations in the real world, and offer potential solutions.

## 1 Introduction

Intergenerational interactions form a rich part of human experience, be it a teacher instructing his pupils, a grandmother reading with her grandson, or a baby boomer and a Gen Z architect collaborating on a building project. The dynamics of how intergenerational social interactions may change over time and their relationship with cognition and wellbeing, from a neuroscientific perspective, are both interesting and challenging to examine. To date, most studies have focused on behavioral, educational, and health-related outcomes of intergeneration interactions (e.g., Knight et al., 2014; Fingerman et al., 2020; Zhong et al., 2020). We take the stance that a deeper understanding of the behavioral and neural mechanisms underlying intra- and inter-generational social interactions will lead to the development and implementation of more effective interventions for social, psychological, and physical wellbeing. The challenge lies in striking the right balance between ecological validity and experimental control. The spontaneity and flow of natural social interaction are quashed by endless questionnaires and cognitive tasks, particularly when such tasks are undertaken in the unnatural and controlled environments required by functional magnetic resonance imaging (fMRI) or magnetoencephalography (MEG). One potential path forward is to use mobile functional near-infrared spectroscopy (fNIRS) to record activity from two or more brains simultaneously, a technique called “hyperscanning” (Montague, 2002; Hasson et al., 2012). Using hyperscanning, we can gain insight into shared patterns of brain activity between two or more people, referred to here as interpersonal neural synchrony (INS).

Here, we first outline the importance of ecologically valid approaches to social interaction research within and between generations, the role that hyperscanning with mobile fNIRS devices can play in advancing this work, and the value of longitudinal observations. Next, we consider potential challenges and future directions associated with mobile intergenerational neuroimaging over repeated sessions. Our aim is to review the state of the art while also signposting areas where cognitive and social neuroscientists may wish to delve further into the social phenomena emerging from intra- and intergenerational dyads.

## 2 Ecologically valid measurements of social interaction

### 2.1 Two-brain neuroscience

The field of social neuroscience has sought to measure behavioral and neural responses to real social interactions *in situ* via an approach called “second-person” or “two-brain” neuroscience (Schilbach et al., 2013). The primary mandate is to ensure that measurements of “social interactions” are in fact gathered from an individual involved in a real, spontaneously unfolding social interaction, rather than observing a pre-recorded social interaction from a third-person perspective. Third-person perspectives of social stimuli, while still common in social neuroscience research, are limited in terms of how much they can inform our understanding of *lived*, as opposed to *observed*, social dynamics (Schilbach et al., 2013; Hasson and Frith, 2016; Redcay and Schilbach, 2019). This first recommendation is couched in a broader recommendation to increase ecological validity, i.e., generalizability to real life, of the experiments we design (Hasson and Honey, 2012; Schilbach et al., 2013; Shamay-Tsoory and Mendelsohn, 2019).

To conduct two-brain neuroscience is to grapple with new ways of maintaining control over the experimental context. One consideration is the added complexity of real-world environments relative to classic lab settings (e.g., sterile, sound-proofed, temperature-controlled, electrically shielded, optimally lit, etc.). For some designs, a table-based conversation or activity may still be run in larger lab spaces, while other designs may require participant pairs to interact within more complex environments. Researchers have adapted to this challenge in different ways. Some researchers generate variable, but highly controlled environments using virtual reality (Park et al., 2018), while others record parallel streams of data from different modalities (e.g., video, motion tracking, eye tracking, heartrate, respiration, etc.), which help researchers link relevant environmental stimulation to changes in brain activity (Kellihan et al., 2013; Pinti et al., 2015, 2018; Ladouce et al., 2022).

The unpredictable nature of spontaneous interaction is also a relevant consideration. For some designs, a trained researcher who adheres to a strict script can curate a specific social context with high consistency across participants (e.g., Lu and Hao, 2019; Moffat et al., 2022). The two-brain approach can enable researchers to record brain activity from each dyad member involved in an interaction to illuminate shared and individual-specific neural activity underpinning the interaction (e.g., Dumas et al., 2011; Dikker et al., 2017; Liu et al., 2021). We will return to the technicalities of hyperscanning–collecting simultaneous neural recordings from two or more people–in Section 3.

### 2.2 Interactive brains between and within generations

As in the opening examples (Section 1), many of our daily activities feature interactions with individuals across generations. Hyperscanning studies commonly investigate social interaction in adult-adult, parent-child, and student-teacher dyads (for reviews see: Wang et al., 2018; Nguyen et al., 2020; De Felice et al., 2023). While the latter two are most strongly represented (Dikker et al., 2022), and involve intergenerational dyads, these are typically limited to young people (i.e., infants, children) paired with adults who have yet to reach middle age. However, many other real-world settings exist where elderly adults interact with younger adults (with their children, in assisted living, when seeking medical care, in community programs) and with children (grandchildren, through volunteer work or community programs). We thus propose that the fields of social and cognitive neuroscience stand to benefit from closer examination of social interactions spanning wider combinations of generational pairings.

What does social neuroscience stand to gain? From a basic science perspective, the field will be enriched by gaining insight into the cognitive and psychological phenomena underpinning social interaction between generations, and whether such interaction can improve social and psychological wellbeing, potentially reducing so-called generational divides (Rubin et al., 2015; Anderson S. et al., 2017; Lokon et al., 2020; Jenkins et al., 2021; Dikker et al., 2022). Further, this work should reveal whether such interactions are supported by shared or individual-specific neural mechanisms (Dikker et al., 2022). From this, we can explore the behavioral and neural mechanisms to maximize social or cognitive outcomes within and across generations. These outcomes, including improving intergenerational communication (Dikker et al., 2022), reducing experiences of loneliness (Tsai et al., 2013; Zhaoyang et al., 2018), identifying social deficits (Schilbach, 2016), and developing interventions (Pan and Cheng, 2020; Chen et al., 2021b), are discussed further in Section 4.

### 2.3 Longitudinal perspectives on interpersonal neural synchrony

To date, hyperscanning studies tend to provide a snapshot of INS (i.e., shared brain activity) between two or more people at one moment in time. Consequently, little is known about how INS *changes* over time. Evidence suggests that the nature of a dyad's relationship may influence INS (Bevilacqua et al., 2019; Djalovski et al., 2021; Dikker et al., 2022; Long et al., 2022), and one could reason that INS may change over the course of a budding relationship. However, many open questions remain, including how INS develops across time and the factors that influence its development (similarity/differences between interactants in terms of age, gender, culture, length, and quality of relationship, etc.).

A handful of studies have collected longitudinal hyperscanning data with a variety of methods, but to date, none have examined changes in INS over time. These have focused on student-teacher and clinician-patient dyads. Dikker et al. (2017) used EEG to measure INS between 12 high-school students in a classroom setting in 11 sessions spread over 3 months, and reported that INS between students reflected their engagement with the lesson. Bevilacqua et al. (2019) measured INS between 12 high-school students and one teacher over six sessions, finding that teacher-student INS was associated with engagement in the lesson and self-reported closeness to the teacher. Neither of these studies (one intergenerational) delved into how INS develops or changes across sessions. Using fMRI, Grahl et al. (2021, 2023) examined clinician-patient INS after six electro-acupuncture treatments for pain across 2 weeks, and promise to present INS results in the future (Ellingsen et al., 2022). Finally, in the *only* longitudinal hyperscanning study operationalizing time, Sened et al. (2022) measured changes in INS between 8 patient-psychotherapist dyads over six therapeutic sessions using fNIRS. Cautioning the need for replication, the authors report increasing INS between therapists and patients over time, as well as an association between increasing INS and reduced symptoms, improved wellbeing and perceived quality of session in inferior frontal gyrus (Sened et al., 2022). Considered together, these studies demonstrate the feasibility of collecting the longitudinal hyperscanning data required to explore the development of INS cross-generationally over repeated interactions.

Researchers studying children's development (Noreika et al., 2020; Turk et al., 2022) and spoken communication (Kelsen et al., 2022) have also signaled that longitudinal hyperscanning could advance their research. Moreover, others suggest that fNIRS would be a suitable technique to address the lack of longitudinal data pertaining to the development of cognition in infants and children (Miguel et al., 2019; Sato et al., 2021), neurodegenerative diseases (Bonilauri et al., 2020; Srinivasan et al., 2023), psychiatric disorders (Kumar et al., 2017; Ho et al., 2020), hearing after receiving a cochlear implant (McKay et al., 2015; Anderson C. A. et al., 2017; Zhou et al., 2022), and use of psychedelic substances (Scholkmann and Vollenweider, 2022). While these authors do not explicitly refer to hyperscanning as their technique of choice, INS recorded with fNIRS over multiple sessions could address several methodological aspirations (i.e., ecological validity, two-brain design, longitudinal assessment) at once.

## 3 Hyperscanning with mobile fNIRS: challenges and paths forward

If we imagine a mother and child are reading a book together, we could record the mother and child's brain activity simultaneously and subsequently assess the similarity of the recorded signal from pre-selected brain areas. Signal similarity is also referred to as coherence, coupling, or synchrony (for review see Scholkmann et al., 2013; Liu and Pelowski, 2014; Minagawa et al., 2018; Czeszumski et al., 2020; Kingsbury and Hong, 2020; Dumas and Fairhurst, 2021; Holroyd, 2022).

Hyperscanning can be achieved with any neuroimaging technique, yet certain techniques lend themselves to studying social interaction better than others: Whereas fMRI and magnetoencephalography (MEG) require participants to be seated or lie down in the scanners, fNIRS and EEG devices are usually easier to position so that interacting individuals can take their natural positions relative to one and other (e.g., Liu and Pelowski, 2014; Dikker et al., 2017). fNIRS is less sensitive to movement than other techniques, making it (more) suitable for measuring brain activity while participants interact (more) naturally (Pinti et al., 2020; Ayaz et al., 2022; Bazán and Edson, 2022; Czeszumski et al., 2022). Even with larger table- or trolley-bound fNIRS systems, fNIRS signals can be recorded while participants engage in movements such as drumming or walking on the spot (Liu et al., 2021; Guérin et al., 2023). The recent advent of mobile fNIRS (lightweight systems with power sources in backpacks, on armbands or entirely encompassed in the cap, as well as lighter cabling or fibreless optodes) are enabling fNIRS recordings during whole body movements in real world environments (e.g., Pinti et al., 2015, 2018; McKendrick et al., 2016; Carius et al., 2020; Joshi et al., 2020; Dybvik and Steinert, 2021).

### 3.1 Recruitment for multi-brain longitudinal studies

Participant attrition over the course of a longitudinal study is to be expected (Matta et al., 2018). Recruitment and attendance challenges may result in missing data from certain individuals or participant pairs, especially when data collection extends over many days or weeks. Researchers must not ignore missing data (Mignogna et al., 2023), which can reduce statistical power and lead to biased parameter estimates if not addressed properly (Matta et al., 2018). Matta et al. (2018) offer evidence-based and scientifically responsible guidelines for dealing with missing data in longitudinal studies. The authors encourage researchers to (1) consider the mechanism causing the missing data (i.e., to consider adding explanatory variables to models), (2) exploit all the available data rather than only complete data sets, and (3) choose an appropriate analysis model (Matta et al., 2018). Krogh-Jespersen et al. (2022) provide additional practical examples of the implementation of these recommendations. The missing values may be estimated using multiple imputation for small amounts of missing data, and interested researchers should take into account the multidimensionality of the missing data before doing so (Molenberghs et al., 2014). To maximize the generalizability of the findings and the benefit of participants' efforts, researchers must plan protocols for addressing missing data, and where possible, include these in the preregistration protocol (Schroeder et al., 2023).

### 3.2 Mobile fNIRS recordings across multiple sessions

During mobile fNIRS recordings in the real world, we must account for environmental factors that may also diminish signal quality (for review see: Pinti et al., 2015, 2018). First, if collecting data outside, excess sunlight could saturate the detectors, but can be avoided using a shading cap made of dark fabric worn over the main cap (Pinti et al., 2015, 2018; McKendrick et al., 2016). Next, warm ambient temperatures could cause the scalp to sweat, leading to absorption of some photons by the sweat (Bronkhorst et al., 2019), the potential for optodes to shift during the recording, and reduced signal quality (Bronkhorst et al., 2019; Perrey, 2022; Doherty et al., 2023). To manage this, study design and session scheduling should account for the expected ambient temperature and added warmth of the fNIRS system with shading cap, as well as the intensity of movements or emotional arousal expected during the recording. Further, extreme body movements could also cause the optodes to shift—particularly when using cabled fNIRS devices. Minimizing such movements via study design and/or participant instructions is advised (Noah et al., 2015; Perrey, 2022).

In a longitudinal context, consistent placement of optodes on the scalp is an aspiration that must be met with reasonable solutions. The optimal scenario would be to position the fNIRS cap according to specific landmarks each time (e.g., Collins-Jones et al., 2021), and to digitize the location of optodes on the scalp (Ayaz et al., 2022). However, where the available equipment, time constraints, or simple biological changes, such as growth, make this impossible, group-level analyses may be the way forward: Collins-Jones et al. (2021) demonstrated with infant data (i.e., head circumference increasing from session to session) that group-level analyses using regions of interest in the channel-space can yield stable estimates. The stability of estimates reflects the level test-retest reliability over time. Studies examining fNIRS test-retest reliability reveal that researchers should incorporate spatial information about location of measurement (Novi et al., 2020; Wu et al., 2021; De Rond et al., 2023) and ensure that physiological components of the signal are handled adequately (Wiggins et al., 2016; Xu et al., 2023).

While a good signal-to-noise-ratio (SNR) is desirable for all fNIRS studies, fNIRS data submitted to INS analyses should have comparable SNRs to reduce the risk of spurious correlations (Noreika et al., 2020). However, when measuring from two or more people, potentially of very different ages, individual differences in hair color and thickness, or scalp thickness may result in substantially different SNRs despite researchers' best efforts. For example, some cultural phenotypes are associated with thicker or darker hair, and it is often the case that young adults tend to have more hair than younger children or older adults, resulting in a noisier signal should this hair slip back under the optodes and obscure the emission of light onto the scalp (Kwasa et al., 2023). In a recent perspective paper, Kwasa et al. (2023) suggest that participant's phenotypic information should be recorded and included in analyses as needed to ensure inclusive sampling in future fNIRS studies. Another consideration is that skull thickness changes with development, with gradual increases in thickness stabilizing from approximately 19 years of age (De Boer et al., 2016; Domenech-Fernandez et al., 2021). This means pairing adolescent (or younger) participants with older partners will likely contribute to differences in SNR (Brigadoi and Cooper, 2015). Calculating the appropriate differential pathlength factor for each participant's age to be used during preprocessing should help (Scholkmann and Wolf, 2013; Nguyen et al., 2021).

### 3.3 Analysis of INS measured with mobile fNIRS

As with most measures of brain activity, INS can be calculated in more than one way. Dumas and Fairhurst (2021) review the different methods, their assumptions, and the type of values they return. However, as a concrete starting point for those wishing to analyze hyperscanning data collected from an intergenerational pair, i.e., parent-child, we recommend beginning with Nguyen et al.'s (2020) guide, for a tutorial of preprocessing steps, wavelet transform coherence (WTC) to obtain INS values, and subsequent group-level analyses in R. Alternatively, see Reindl et al. (2019) or Hu et al.'s (2020) video guides, as well as the purpose-built HyPyP package (Ayrolles et al., 2021).

Regardless of one's preferred method of calculating INS, the first step is to preprocess the fNIRS data per individual participant. Optical intensity signals are first converted to optical density using an age-appropriate differential pathlength factor (Scholkmann and Wolf, 2013; Nguyen et al., 2021). Next, motion artifacts are corrected using algorithms built into one's chosen analysis software, and the SNR may be subject to a threshold (although no standard value exists for the field). For real-world data collected while participants move freely, visual inspection of the signal is particularly important to ensure that motion artifacts are truly minimized (Pinti et al., 2018). Given the importance of motion correction, we refer readers to Huang et al. (2022) review of algorithms for motion correction, as well as the role of accelerometers in correcting head motion, or Delgado Reyes et al. (2018) comparison of algorithms implemented on children's data. Participant age is a relevant consideration when choosing a motion correction algorithm, as infants and children tend to make more frequent and larger movements than adults, meaning that certain algorithms may be more suitable than others (Di Lorenzo et al., 2019; Fishburn et al., 2019; Hu et al., 2020). After correcting for motion artifacts, the signal is typically subjected to filtering (e.g., a bandpass filter between 0.01 and 0.5 Hz) to minimize physiological confounds and, finally, converted to concentrations of oxygenated and deoxygenated hemoglobin using the modified Beer-Lambert law (Hu et al., 2021; Nguyen et al., 2021). Physiological or systemic confounds also require substantial consideration. Best practice is to use short channels (i.e., channels <15 mm) wherever possible to isolate and regress out extracerebral signal components (Yücel et al., 2021). Heart-rate and respiration can also be recorded, for example using a heart-rate monitor and a respiration belt, and regressed out of the fNIRS signal, demonstrably yielding an fNIRS signal with fewer systemic confounds (Scholkmann et al., 2022). For further details on filter selection and the order in which these steps can and should be applied, we refer interested readers to a review by Pinti et al. (2019) and an associated commentary (Bizzego et al., 2020).

For fNIRS data, WTC is a commonly used measure of INS, as it is not strongly influenced by differences in the shape of the hemodynamic response between individuals or brain regions (Sun et al., 2004). Correlations between participants (e.g., Nastase et al., 2019) may show high correlations where little to no change is measured, offering misleading estimates of INS for those regions (Nguyen et al., 2021). To explore questions such as “whose brain is leading, or shows the pattern of activation first, and at which latency is the other brain following?”, one may consider time-shifting analyses such as Granger causality (Leong et al., 2017; Dumas and Fairhurst, 2021). Exploring potential changes in leader-follower dynamics will also be relevant to gain a full understanding of the development of intergenerational interactions. Regardless which measure of INS is selected, it is advisable to calculate INS for shuffled dyads who never interacted, thereby removing any components of the INS value stemming from true social interaction (Hirsch et al., 2021; Nguyen et al., 2021). This yields a “baseline” or “chance level” of INS with which INS in real dyads can be contextualized.

Finally, we would like to urge that these processes be embedded in open science practices (Ayaz et al., 2022; Kelsey et al., 2023), beginning with preregistration where explicit intentions are established for the research prior to collecting data (for recommendations see: Schroeder et al., 2023). Following analysis, datasets should be publicly archived to promote transparency and re-use of data. These small steps will allow future work to build upon previous methods and findings more easily while also addressing current challenges facing hyperscanning research (Kelsey et al., 2023).

### 3.4 Interpreting changes in INS in longitudinal intergenerational datasets

Responsible data interpretation requires well-designed experiments to isolate social features or dynamics of interest. If not carefully controlled for, measures of INS can reflect the communal perception of external environmental changes or perturbations (Czeszumski et al., 2020; Hamilton, 2021; Holroyd, 2022) rather than synchrony stemming from the inter-brain mechanisms under examination. Researchers should employ an active control condition (i.e., not resting state) that matches the test condition as closely as possible to enhance the likelihood of uncovering mechanistic explanations for synchrony (Moreau and Dumas, 2021; Novembre and Iannetti, 2021). For example, comparisons of INS while participants complete the task together and alone, or between conditions such as verbal agreement and disaggreement (Hirsch et al., 2021) are preferable to an active condition vs. resting state. Researchers should additionally account for each dyad's social closeness and the amount of eye contact, as both can influence synchronization of spontaneous cortical activity and could be epiphenomenal confounds to targeted explanatory phenomenon (e.g., Bevilacqua et al., 2019; Djalovski et al., 2021; Dikker et al., 2022; Guglielmini et al., 2022; Long et al., 2022; Koul et al., 2023). Social closeness may be measured using behavioral scales, such as the inclusion of self-in-other scale (Aron et al., 1992), for example. Eye contact could be explicitly controlled using physical barriers or, perhaps more elegantly, recorded using eye-tracking glasses and included in the analysis of fNIRS signal (Hirsch et al., 2017).

As intergenerational perspectives on INS remain limited, the influence of physiological, social, and cognitive changes relating to maturation on INS remain unknown. Dikker et al. (2022) predict that changes in cognitive processing speeds over the lifespan may evoke forms of interpersonal compensation. We agree with Dikker and colleagues and suggest that this compensation is likely represented in INS, and that careful consideration should be given to how this information can be quantified, to establish field standards. Further, the interpretation of data collected in longitudinal hyperscanning studies, particularly with an intergenerational focus, stands to be enriched by weaving both qualitative and quantitative measures into the design (Deschepper et al., 2017; Dikker et al., 2021).

## 4 Future directions

### 4.1 Socially-centered interventions

Aligned with the concept of two-brain neuroscience, insight into shared and idiosyncratic aspects of neural activity in the context of everyday social interactions also hold promise for elucidating potential origins of atypical processing in some disorders (Schilbach, 2016; Redcay and Schilbach, 2019). Further, changes in INS may be useful to benchmark progress in interventions for social, psychological, and physical wellbeing. As Sened et al. (2022) recently demonstrated, changes in patient-therapist INS can be observed across as few as six psychotherapy sessions. While this study remains a proof of concept, warranting replication with a greater sample size, similar studies could probe INS between clinicians and patients experiencing a wider variety of ailments (e.g., chronic pain, loneliness, anxiety, and perhaps even reading difficulties or cognitive deficits, etc.) or between these patients and their primary caregivers or frequent social contacts (Kruppa et al., 2021; Short et al., 2021; Deng et al., 2022; Tucek et al., 2022; Provenzi et al., 2023; Wei et al., 2023). The latter would be instrumental for family-centered and community-based care (Short et al., 2021; Provenzi et al., 2023).

Community care, interpreted more broadly, can include social programs promoting wellbeing across the lifespan. Some evidence suggests that intergenerational activities enhance social cohesion between generations and older individuals' wellbeing, with enhancement accruing over repeated interactions (Rubin et al., 2015; Anderson S. et al., 2017; Lokon et al., 2020; Jenkins et al., 2021). Similarly, programs that pair youths with non-parent mentors can positively influence youths' emotional wellbeing, resilience, as well as academic and career achievement (Van Dam et al., 2018; Raposa et al., 2019; Goldner and Ben-Eliyahu, 2021). A neural perspective on the development of such intergenerational relationships could help concretize their value, providing an additional stream of empirical evidence. In addition to learning about INS over time, we could potentially offer these intergenerational dyads a readout of how “in sync” their brains are–a novel activity with the potential to enrich relationship development.

### 4.2 Neurofeedback

An emerging technique called “neurofeedback” could also be integrated with fNIRS hyperscanning data (Kohl et al., 2020). Neurofeedback involves giving explicit feedback about brain activity to the participant in real time with the aim of assessing changes in behavior and/or brain function (Thibault et al., 2016; Orndorff-Plunkett et al., 2017; Sitaram et al., 2017; Kadosh and Staunton, 2019). We propose that real-time INS between a patient and relevant partner (e.g., clinician, family member, support person) could be presented to the dyad as the stimulus for learning or changing social behaviors. The success, or potential lack of success, of hyperscanning neurofeedback studies would open new avenues for studying social learning.

Dikker et al. (2021) describe a particularly elegant implementation of hyperscanning neurofeedback, run over 5 years in museums, wherein participants sat face-to-face in chairs with large canvas shells behind them, and the relative degree of a dyads' INS was projected on the canvas in real time. In a sample of 363 dyads, the authors demonstrated that explicitly cueing participants to the degree or changes in their INS using a light display could increase INS, plausibly as a result of increased motivation and attention (Dikker et al., 2021). Further research using hyperscanning to provide neurofeedback has also demonstrated success in guiding behavior (Duan et al., 2013; Chen et al., 2021a,b; Müller et al., 2021), albeit always within same generation dyads. As an aside, Dikker et al. (2019) operationalized neurofeedback using EEG to bring generations together in the form of interactive art installations–a possible avenue yet to be explored with mobile fNIRS.

To date, only Duan et al. (2013) have used fNIRS hyperscanning in a neurofeedback paradigm, in which they demonstrate the feasibility of fNIRS in a dual-brain neurofeedback loop. Mobile fNIRS devices have yet to be used in hyperscanning neurofeedback settings, but should ensure participants' comfort and the feasibility of integrating neurofeedback into real world settings and social interactions (Nazneen et al., 2022). Given the portability and suitability of mobile fNIRS devices for use with all populations (Ayaz et al., 2022), we expect to see more neurofeedback interventions involving two or more brains embedded in the real world, while keeping ethical concerns about the use of such technologies front and center (UNESCO, 2022). Further we recommend that future research explores the value of neurofeedback in intergenerational dyads, such as parent-child or clinician-patient (child or senior) dyads, to determine the true generalizability of such tools in clinical practice.

## 5 Concluding remarks

The increasing availability of mobile fNIRS devices alongside the growing technical knowledgebase offers a promising avenue for investigating the development of INS over time. We encourage researchers to take advantage of these to help characterize the mechanisms underpinning social interactions within and across generations.

## Data availability statement

The original contributions presented in the study are included in the article, further inquiries can be directed to the corresponding author.

## Author contributions

RM: Conceptualization, Writing – original draft, Writing – review & editing. CC: Writing – original draft, Writing – review & editing. EC: Conceptualization, Writing – review & editing.
